# Subfunctionalization of D27 Isomerase Genes in Saffron

**DOI:** 10.3390/ijms231810543

**Published:** 2022-09-11

**Authors:** Alberto José López-Jiménez, Lucía Morote, Enrique Niza, María Mondéjar, Ángela Rubio-Moraga, Gianfranco Diretto, Oussama Ahrazem, Lourdes Gómez-Gómez

**Affiliations:** 1 Instituto Botánico, Departamento de Ciencia y Tecnología Agroforestal y Genética, Universidad de Castilla-La Mancha, Campus Universitario s/n, 02071 Albacete, Spain; 2 Escuela Técnica Superior de Ingenieros Agrónomos y Montes, Grado de Biotecnología, Departamento de Ciencia y Tecnología Agroforestal y Genética, Universidad de Castilla-La Mancha, Campus Universitario s/n, 02071 Albacete, Spain; 3 Facultad de Farmacia, Departamento de Ciencia y Tecnología Agroforestal y Genética, Universidad de Castilla-La Mancha, Campus Universitario s/n, 02071 Albacete, Spain; 4 Italian National Agency for New Technologies, Energy, and Sustainable Development, Casaccia Research Centre, 00123 Rome, Italy

**Keywords:** apocarotenoids, carotenoids, expression, isomerase activity, leaves, mycorrhiza, root, stigmas

## Abstract

Chromoplasts and chloroplasts contain carotenoid pigments as all-*trans*- and *cis*-isomers, which function as accessory light-harvesting pigments, antioxidant and photoprotective agents, and precursors of signaling molecules and plant hormones. The carotenoid pathway involves the participation of different carotenoid isomerases. Among them, D27 is a β-carotene isomerase showing high specificity for the C9-C10 double bond catalyzing the interconversion of all-*trans*- into 9-*cis*-β-carotene, the precursor of strigolactones. We have identified one *D27* (*CsD27-1*) and two *D27-like* (*CsD27-2* and *CsD27-3*) genes in saffron, with *CsD27-1* and *CsD27-3*, clearly differing in their expression patterns; specifically, *CsD27-1* was mainly expressed in the undeveloped stigma and roots, where it is induced by *Rhizobium* colonization. On the contrary, *CsD27-2* and *CsD27-3* were mainly expressed in leaves, with a preferential expression of *CsD27-3* in this tissue. In vivo assays show that CsD27-1 catalyzes the isomerization of all-*trans*- to 9-*cis*-β-carotene, and could be involved in the isomerization of zeaxanthin, while CsD27-3 catalyzes the isomerization of all-*trans*- to *cis*-ζ-carotene and all-*trans*- to *cis*-neurosporene. Our data show that CsD27-1 and CsD27-3 enzymes are both involved in carotenoid isomerization, with CsD27-1 being specific to chromoplast/amyloplast-containing tissue, and CsD27-3 more specific to chloroplast-containing tissues. Additionally, we show that *CsD27-1* is co-expressed with *CCD7* and *CCD8* mycorrhized roots, whereas *CsD27-3* is expressed at higher levels than *CRTISO* and *Z-ISO* and showed circadian regulation in leaves. Overall, our data extend the knowledge about carotenoid isomerization and their implications in several physiological and ecological processes.

## 1. Introduction

Carotenoids are a class of C40 hydrocarbon compounds formed through the condensation of isoprenoids [1]. They play important roles in numerous physiological processes in plants. In more detail, carotenoids act as accessory pigments in photosynthesis and serve as photoprotective agents by quenching singlet oxygen, which might damage chlorophyll. In addition, carotenoids act as precursors in the biosynthesis of apocarotenoids, such as vitamin A and abscisic acid (ABA) or strigolactones (SLs) [1]. In particular, SLs are carotenoid-derived terpenoid lactones that were for the first time identified as germination stimulants for parasitic plant seeds [2]. Later on, SLs were shown to affect different aspects of plant biology: they act as signals to recruit arbuscular mycorrhizal fungi, and more recently as plant hormones to regulate different processes, e.g., plant architecture (inhibition of bud outgrowth and of shoot branching), root development, stem growth, senescence [3], responses to abiotic factors [4] and flower induction [5].

While all-*trans*-β -carotene acts as a precursor to SLs, *trans*-zeaxanthin has also been shown to act as a precursor to other non-canonical SLs [6]. Both carotenoids are converted sequentially into carlactone and 3-OH-carlactone, respectively, by the isomerase D27, followed by the action of carotenoid cleavage dioxygenase 7 (CCD7) and 8 (CCD8) [7], which have been previously characterized in saffron [8]. Further conversion of carlactone to carlactonic acid is catalyzed by cytochrome P450 monooxygenase More Axillary Growth 1 (MAX1/CYP711A in the P450 family) [7].

**Figure 1:**
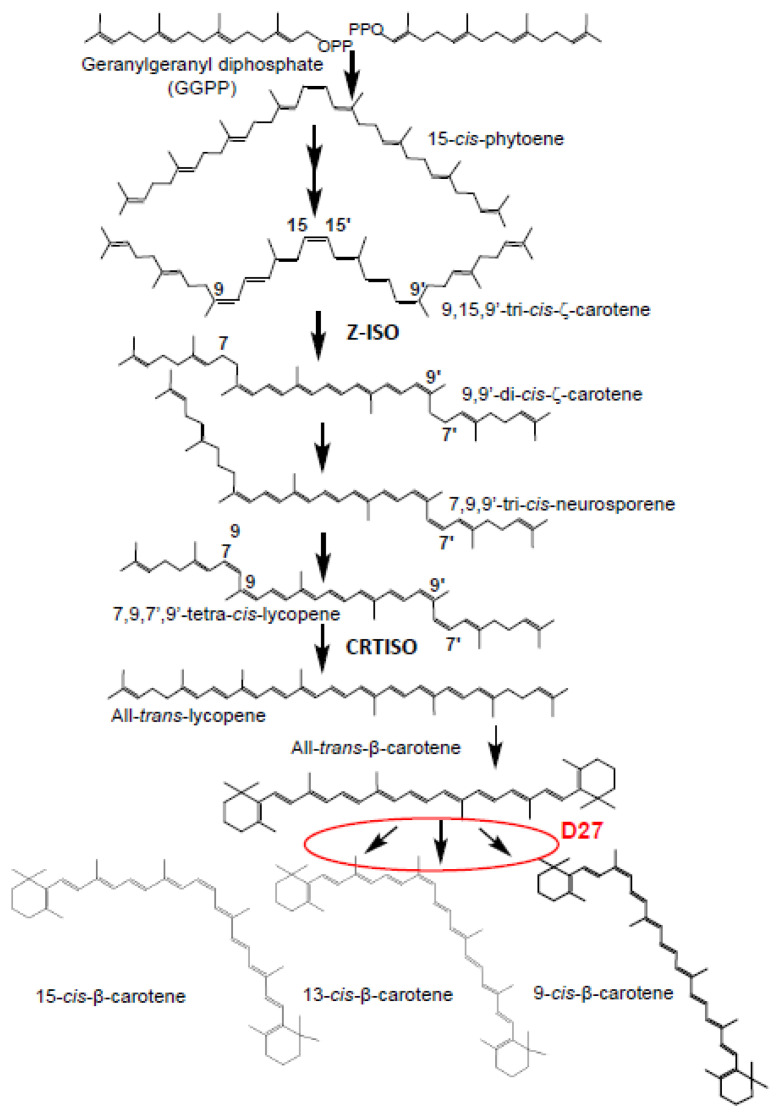
Carotenoid biosynthetic pathway from geranylgeranyl diphosphate to β-carotene. The steps in which carotenoid isomerases are known to be involved are indicated by the name of the corresponding enzymes. The described isomerase activity of D27 over β-carotene is indicated in red.

D27 was originally mutationally defined in rice [9], and reverse genetic approaches in Arabidopsis indicate a similar function in these species [10]. D27 is an iron-containing protein with isomerase activity that produces the substrates for CCD7 [7] (Figure 1). In plants, green algae, and cyanobacteria, the D27 family is divided into three clades: clade 1, clade 2 and clade 3. The first described D27 protein, involved in SL biosynthesis, belongs to clade 1. The function of the other D27 homologs remains unknown [10], albeit the presence of reduced levels of ABA in loss-of-function mutants of these D27 homologs suggest their implication in the isomerization of neoxanthin or violaxanthin used in ABA biosynthesis [11]. Although Arabidopsis *D27* is induced by ABA [12], the mutant also showed reduced ABA levels, but is not involved in the formation of ABA precursors, 9-*cis*-violaxanthin or -neoxanthin [13]. However, several studies suggest a connection between the SL and ABA pathways; ABA-deficient mutant plants impaired in different steps of ABA biosynthesis exhibit reduced SL levels, and SL mutants have altered ABA levels [14,15]. Interestingly, another carotenoid isomerase, Ζ-ISO, which functions in isomerization of 9,15,9′-tri-*cis*-ζ-carotene to 9,9′-di-*cis*-ζ-carotene (Figure 1), and CRTISO, which catalyzes isomerization of 7,9,9′,7′-tetra-cis-lycopene or prolycopene to all-*trans*-lycopene (Figure 1), have been shown to be required for the production of SLs and ABA in rice [16,17].

*Crocus sativus* L. is an economically important monocotyledonous crop producing saffron spice obtained from the dry stigmas of the flower. *C. sativus* is a sterile plant, multiplied through a subterranean modified stem, the corm [18]. The appreciation for saffron spice as a food additive has been observed in the past and continues today, being considered the world’s highest priced spice [19]. In addition, saffron continues to be used in the traditional medicine of many cultures [20,21]. Numerous studies have demonstrated its therapeutic properties, [22,23,24,25] increasing the demand of saffron for medical and cosmetic applications. Therefore, it will be necessary to develop new methodologies in order to increase the production of this crop. Altitude, temperature, photoperiod, soil, and topographical location are the critical environmental parameters that affect saffron production [26]. In this context, strigolactones, as important players in plant growth and development and also in plant adaptation to environmental changes [27], can pave the way for new innovative crop enhancement applications. However, few studies in saffron have focused on SLs and their related processes: we have previously displayed that CsCCD7 and CsCCD8 are involved in the production of SLs which take part, synergistically with auxins, in the inhibition of corm axillary bud sprouting [8], and that phytoene synthase 3 (*CsPSY3*) expression is associated with mycorrhizal colonization and strigolactone synthesis [28].

Here, we isolated and characterized three *D27* members, the canonical *D27* gene in saffron (*CsD27-1*) and two *D27-like* genes (*CsD27-2* and *CsD27-3*). *CsD27-1*, the functional ortholog of rice *DWARF27*, was mainly expressed in stigma and in roots, where it was induced by *Rhizobium* colonization. By contrast, *CsD27-2* and *CsD27-3* were mainly expressed in leaves, with *CsD27-3*, showing a much higher expression in this tissue exhibiting, additionally, a diurnal expression pattern. While the CsD27-2 function remains unknown, CsD27-3 catalyzed the isomerization of linear carotenoids ζ-carotene and neurosporene, acting in the opposite direction to the carotenoid isomerases Z-ISO and CRTISO.

## 2. Results

### 2.1. Isolation and Identification of SL Biosynthetic Gene D27 in Saffron

**Figure 2:**
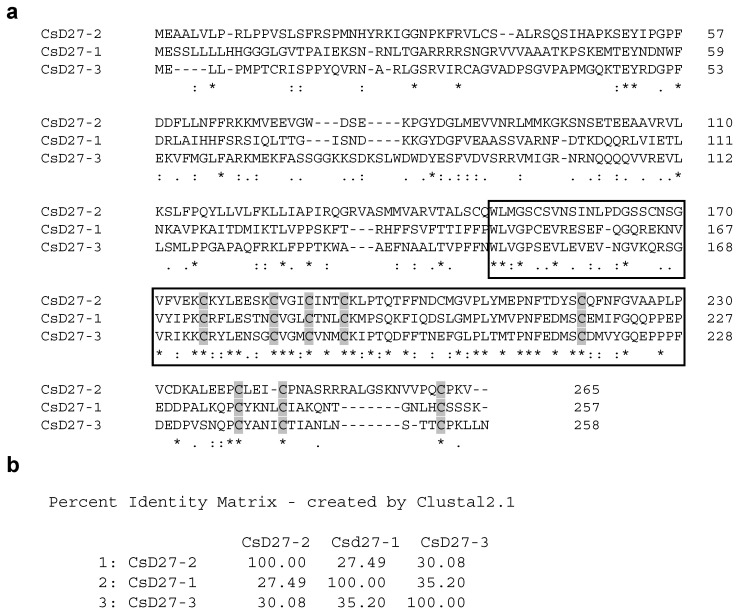
Sequence characteristics of CsD27 enzymes. (**a**) Amino acid sequence alignments among CsD27 enzymes. The amino acid sequence alignments were made by CLUSTAL omega. Conserved cysteines (C) are highlighted in grey, to indicate structural conservation. Amino acids that constitute the conserved domain of D27 isomerase in higher plants are framed in black. Asterisks denote fully conserved residues, while colons and dots denote partially conserved residues. (**b**) Percent identity matrix among the CsD27 amino acid sequences.

**Figure 3:**
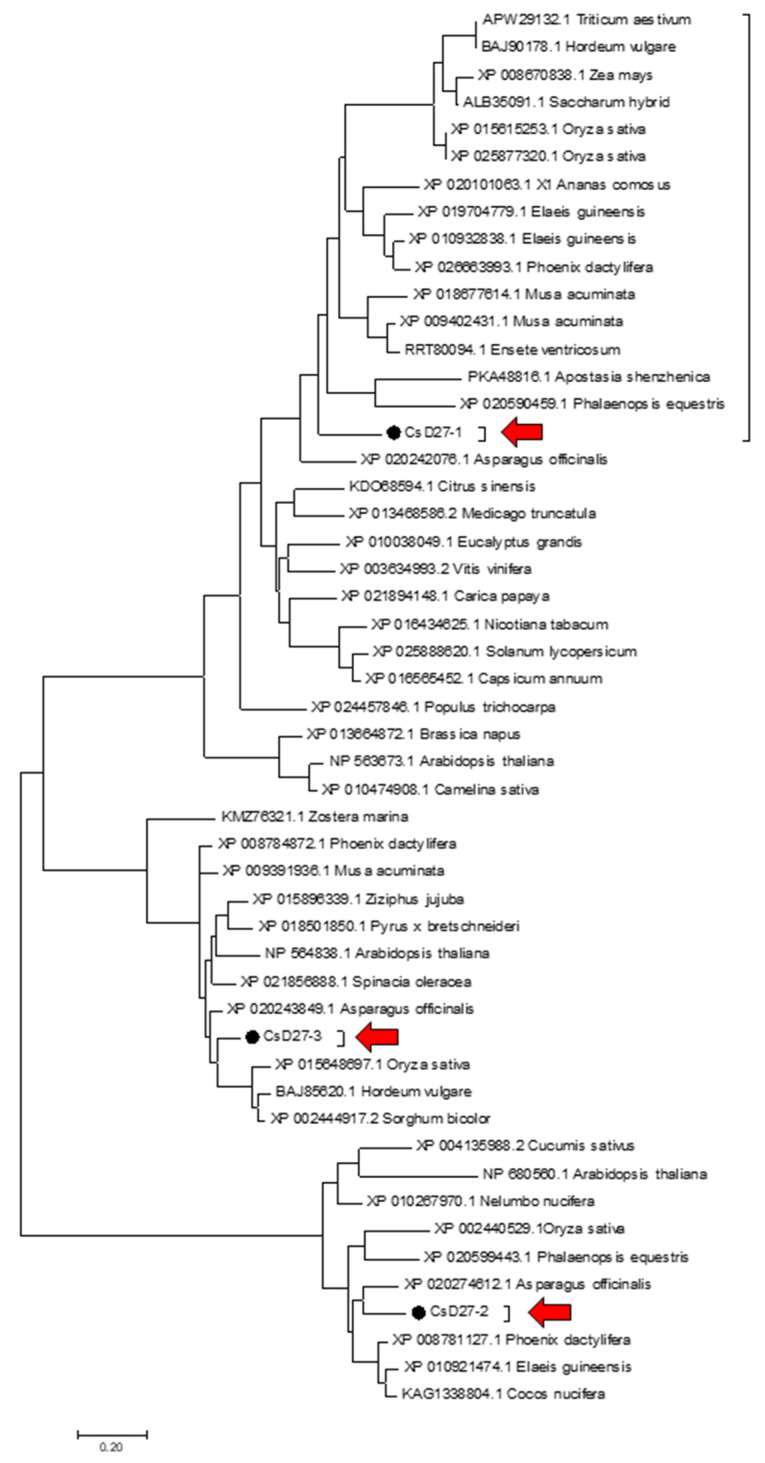
Phylogenetic tree of the CsD27 amino acid sequences with other plant members of the D27 family. The unrooted phylogenetic tree was constructed using MEGA7 from the D27 sequences retrieved from the GenBank database. Evolutionary relationships were inferred using the Neighbor-joining method with 2500 bootstrap re-sampling strategy. The D27 sequences from saffron are highlighted by black dots and red arrows.

We used the amino acid sequences of rice and Arabidopsis D27 proteins as queries to identify putative D27 orthologs in saffron transcriptomes [18,29]. Three D27 homologues, all containing the DUF4033 domain, were identified, and named as CsD27-1 (GenBank ON003971), CsD27-2 (GenBank ON003972) and CsD27-3 (GenBank ON003973) (Figure 2a). The identity among the amino acid sequences ranged from 27–35% (Figure 2b). *CsD27-1* encodes a protein of 257 amino acids in length and showed the highest identity (61.5%) of XP_020242076.1 from *Asparagus officinalis*, with the major differences being present in the first N-t part of the amino acid sequence. CsD27-2 showed the highest identity (71%) of XP_010921474.1 from *Elaeis guineensis*, and CsD27-3 showed the highest identity (80%) of XP_008784872.1. from *Phoenix dactylifera*. The prediction program DeepLoc (http://www.cbs.dtu.dk, accessed on 5 December 2021) predicts a transit peptide for plastid location for all the analyzed CsD27 proteins, in addition to association to membranes (Figure S1). Subsequently, the amino acid sequences for CsD27-1 to -3 were used to build up a phylogenetic tree to determine their positions among other D27 proteins from different plant species (Figure 3). The CsD27 proteins fall into three different clusters (Figure 3); depending on the plant species, up to three D27 paralogs have been identified. This is the case in Arabidopsis, rice [10], maize, apple, strawberry [30], *E. guineensis*, and *Musa accuminata*. However, in these species and in most of the plant species sequenced so far, only one canonical copy for *D27* is present in the genome (https://phytozome.jgi.doe.gov/, accessed on 2 April 2021), and the other genes are referred to as *D27-like* [10]. In saffron, *CsD27-1* and *CsD27-3* seem to have evolved by gene duplication from a single *D27-like* gene copy (Figure 3), while *CsD27-2* evolved independently.

### 2.2. Expression Levels in Vegetative Tissues and Saffron Stigmas

**Figure 4:**
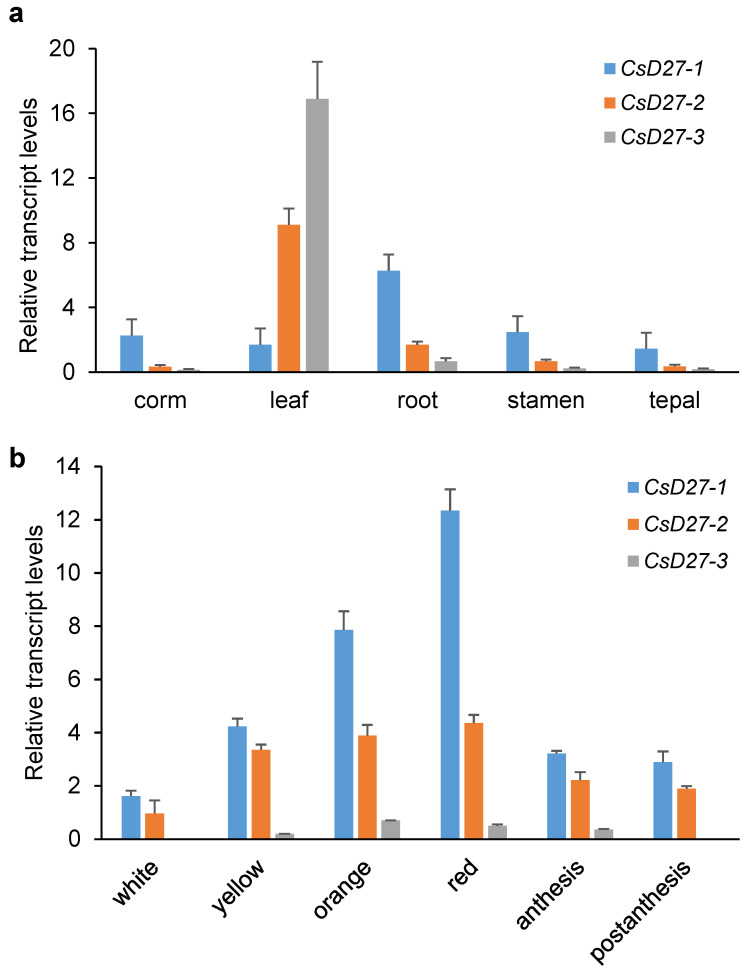
Relative expression levels of *CsD27-1*, -*2* and -*3* in vegetative and reproductive tissues investigated by qRT-PCR. (**a**) Transcripts levels in corm, leaf, root, stamen and tepal. (**b**) Expression levels in six developmental stages of the stigma. Bars represent mean ± SD (*n* = 3 biological replicates).

To determine the possible roles of these enzymes in saffron, their expression levels were analyzed by qPCR in different vegetative tissues and in the stigma throughout its development. Among all tissues tested, *CsD27-1* was preferentially expressed in the roots, while *CsD27-2* and *CsD27-3* were mainly expressed in the leaves (Figure 4a). The expression patterns were also analyzed in the stigma tissue at different developmental stages. In this tissue, *CsD27-1* transcripts increased from the yellow to the red stages, and the expression decreased afterwards (Figure 4b). *CsD27-2* was mainly expressed in the undeveloped stigma, but at lower levels than in leaves, and *CsD27-3* showed low expression levels in the stigma tissue (Figure 4b).

We then further investigated the expression profiles of *CsD27-1* and *CsD27-3*, due to their predominant expression in specific tissues, in roots for *CsD27-1* and leaves in the case of *CsD27-3*. The expression of *CsD27-1* was analyzed in mycorrhized and non-mycorrhized roots, in addition to the expression analyses of *CCDs*, including *CCD1*, *CCD4*, *CCD7* and *CCD8*. The levels of expression of *CsD27-1*, *CCD7* and *CCD8* were higher in mycorrhized roots than in non-mycorrhized roots (Figure S2a). For *CsD27-3*, its expression level was analyzed in leaves at different time points throughout the day. We noticed fluctuations of its expression level: it was highly expressed between 10:00 till 14:00, and its expression dropped between 22:00 and 02:00. (Figure S2b).

Simultaneously, we analyzed the carotenoid profiles in stigmas, leaves and roots (Figure S3 and Table S2), giving special consideration to *cis*-isomers. In the stigma tissue, zeaxanthin and all-*trans*-β-carotenes were the main carotenoids detected (Figure S3 and Table S2), whereas in leaves, all-*trans*-β-carotene, lutein and all-*trans*-violaxanthin were the most abundant carotenoids (Figure S3 and Table S2). In roots, the main carotenoid detected was all-*trans*-β-carotene (Figure S3 and Table S2). Regarding the presence of *cis*-isomers in these tissues, leaves showed the highest number of *cis*-carotenoids, mainly *cis*-violaxanthin and *cis*-lutein isomers (Table S2), which were absent in stigma and root tissue. In stigma tissue, *cis*-isomers were detected for lycopene, which was absent in leaves and root tissue. However, *cis*-isomers of β-carotene were detected in roots, stigma, and leaves (Table S2). We further analyzed the expression of the carotenoid isomerases *ZISO* and *CRTISO* in stigma, in dark (D) and light (L), and in leaves (Figure S4). The expression levels of both isomerases were very high in the stigma tissue, much higher than the expression of *CsD27-1*, but were present at much lower levels in leaves, where *CsD27-3* was expressed at higher levels (Figure S4).

### 2.3. Activity Assays of CsD27 in E. coli and N. benthamiana

**Figure 5:**
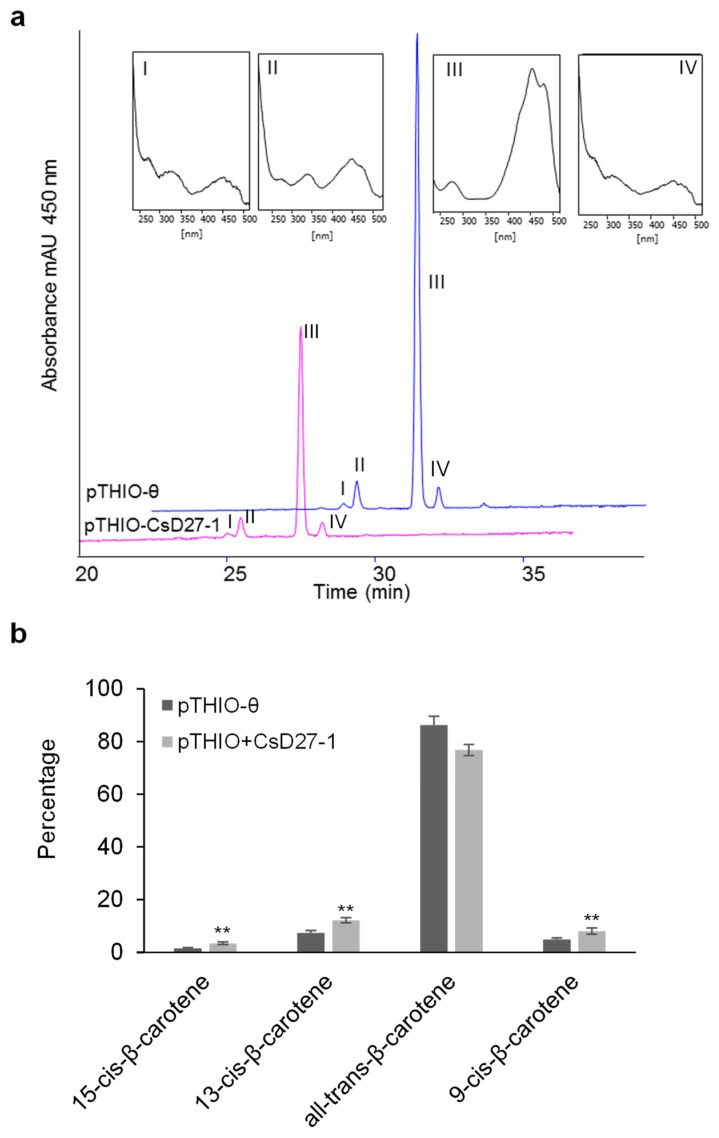
HPLC analysis of the reaction catalyzed by CsD27-1 in vivo on β-carotene produced by *E. coli* cells. (**a**) pThio+CsD27-1, converts all-*trans*-β-carotene (III) to 9-*cis*-β-carotene (IV), 13-*cis*-β-carotene (II) and to15-*cis*-β-carotene (I). (**b**) Percentages of all the *cis*-β-carotene isomers were significantly increased in the cells expressing the CsD27-1 enzyme. Statistical analysis was performed using student-t test. A designation of ** = *p* < 0.01 (*t*-test). *n* = 3 independent biological replicate experiments. Error bars represent SD.

Carotenoid-accumulating *E. coli* cells have been used to characterize the activity of rice OsD27 and Arabidopsis D27 [13,31]. We focused our interest in CsD27-1 and CsD27-3, due to their tissue-specific expression, and their activities were tested using *E. coli* cells accumulating different carotenoids. *CsD27-1* was expressed in β-carotene accumulating *E. coli* strains to investigate whether the saffron enzyme showed the same activity previously determined for the rice and Arabidopsis enzymes on β-carotene. The introduction of *CsD27-1* in β-carotene-accumulating *E. coli* cells led to an increase in 13-*cis*, 15-*cis* and 9-*cis*: all-*trans*-β-carotene ratio, compared to the control extracts obtained from *E. coli* cells, transformed with the void plasmid (Figure 5a,b).

**Figure 6:**
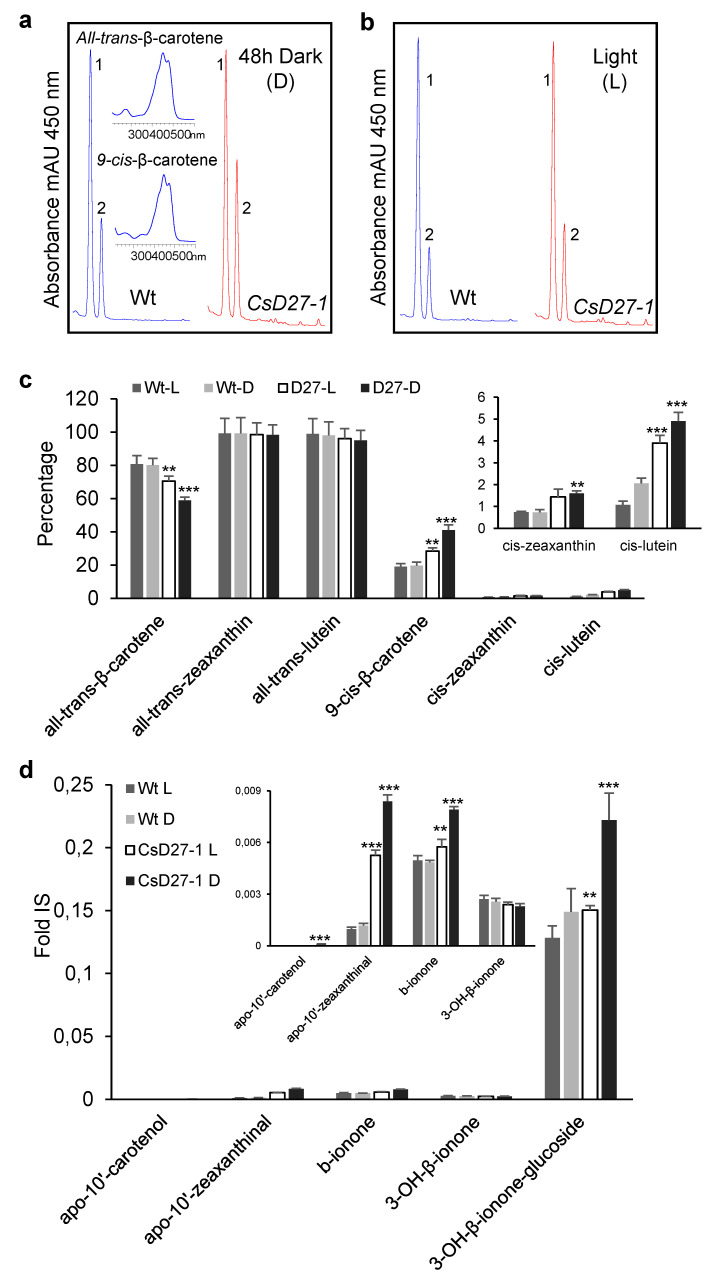
In planta activity of CsD27-1. (**a**) HPLC analysis of in vivo CsD27-1 activity in leaves of *N. benthamiana* plants grown during 48 h in dark conditions. (**b**) HPLC analysis of in vivo CsD27-1 activity in leaves of *N. benthamiana* plants grown under standard light conditions. (**c**) Percentage of cis-9-β-carotene isomer was significantly increased in *N. benthamiana* leaves agroinfiltrated with *35S::CsD27-1* in both dark and under standard light conditions. Ratio was calculated based on peak area of 9-*cis*-β-carotene to all-*trans*-β-carotene in the chromatograms in (**a**,**b**). (**d**) Increase in apocarotenoid levels in *N. benthamiana* leaves agroinfiltrated with *35S::CsD27-1* in both dark and under standard light conditions. Statistical analysis was performed using the Student *t*-test. Asterisks indicate differences from wild type (** = *p* < 0.01, *** = *p* < 0.001 *t*-test). *n* = 3 independent biological replicate experiments. Error bars represent SD.

The observed activity in β-carotene-accumulating *E. coli* was further tested by transient expression of *CsD27-1* in *Nicotiana benthamiana* leaves. To optimize the accumulation of CsD27-1 in *N. benthamiana* leaves, the gene was transiently co-expressed in 4-week-old wild-type *N. benthamiana* plants together with the gene *P19*, encoding a viral gene silencing suppressor from the *Cymbidium* ringspot virus (Pbin61:P19 vector). Two batches of plants were prepared; one batch was kept 8 dpi under normal growth conditions (12 h of light per day, 24 °C, 60% relative humidity), and the other batch of plants was grown in the dark in the last 48 h before the material was collected at 8 dpi. The analyses of β-carotene isomers revealed an increase of *cis*-isomers, higher in the dark conditions (Figure 6a–c). An increase was also observed of *cis*-isomers of zeaxanthin and lutein (Figure 6c), although the percentage of these *cis*-isomers was lower compared with the *cis*-isomers of β-carotene. Further, we analyzed these samples for the presence of apocarotenoids that could result from the activity of CCD7 on β-carotene isomers (Figure 6d). CCD7 catalyzed the cleavage of 9-*cis*-β-carotene, leading to 9-*cis*-β-apo-10′-carotenal and β-ionone [32]. Similarly, it also catalyzed the cleavage of the 9′,10′ double bond in 9-*cis*-zeaxanthin, leading to 9-cis-3-OH-β-apo-10′-carotenal and 3-OH-β-ionone [33]. We searched for the presence of apocarotenoid compounds in the tobacco leaves from the transient expression assays. Several apocarotenoids were detected at different levels in the analyzed samples, with the highest levels detected in those obtained from the leaves transiently expressing *CsD27-1* (Figure 6d). These leaves also showed higher levels of ABA and ABA glucosyl ester (ABA-GE) compared to the other samples (Figure S5).

Due to the detection of higher levels of apocarotenoids derived from the zeaxanthin cleavage in the samples expressing *CsD27-1*, the gene was introduced in zeaxanthin-accumulating *E. coli* cells to test its activity on all-*trans*-zeaxanthin, but no isomerization was observed (Figure S6).

**Figure 7:**
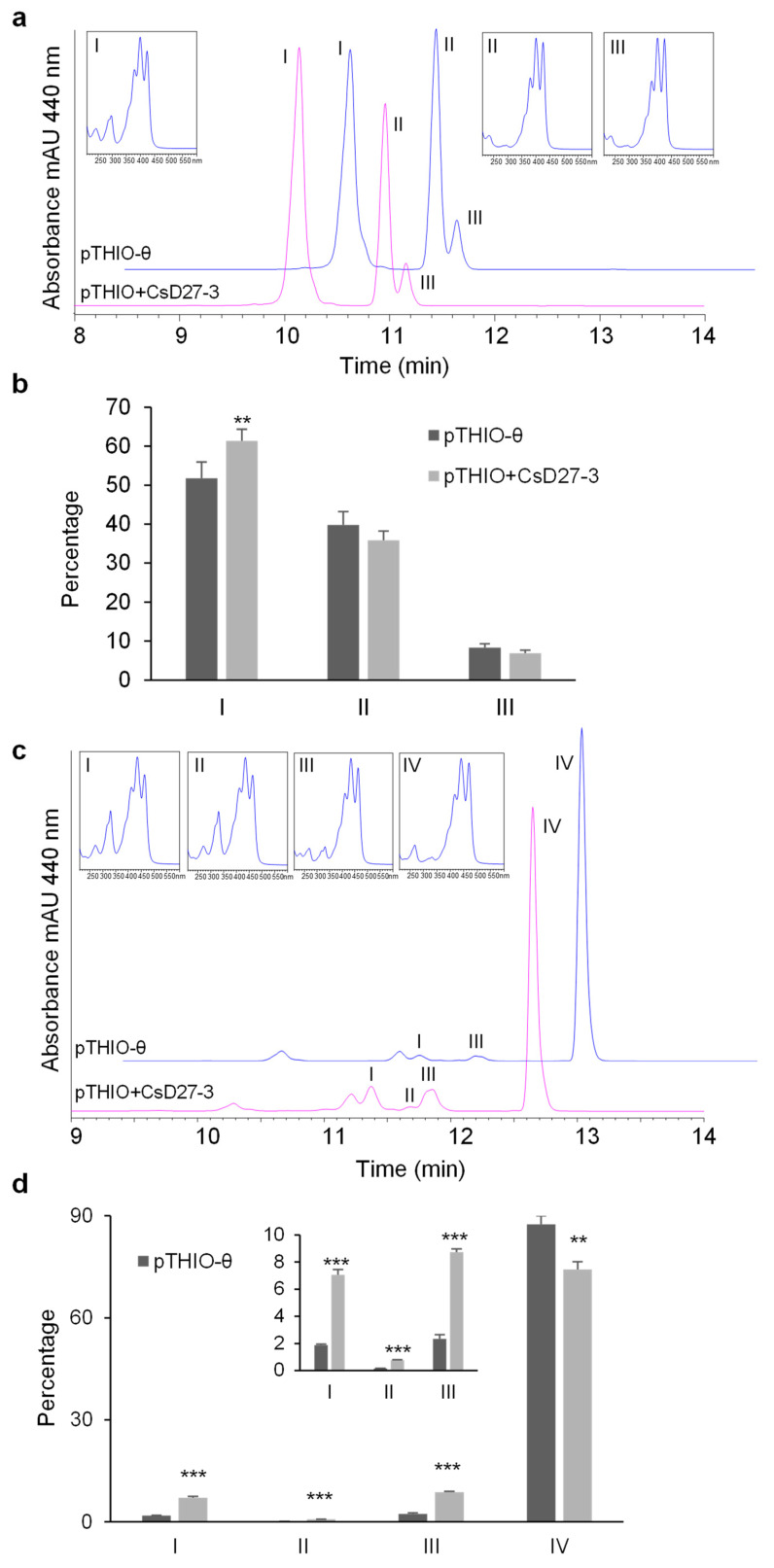
Analysis of CsD27-3 activity in *E. coli* cells accumulating the linear carotenoids ζ-carotene and neurosporene. (**a**) HPLC analyses of carotenes extracted in the absence (pThio-θ) and presence (pThio+CsD27-3) of CsD27-3. MaxPlot chromatograms showing each peak at its spectra are presented. Peak I: 9,15,9′-tri-*cis*-ζ-carotene; peak II: 9,9′-di-*cis*-ζ-carotene; peak III: all-*trans*-ζ-carotene. Absorption spectra of specific peaks are presented in inset boxes. 9,15,9′-tri-*cis*-ζ-carotene is distinguished from the 9,9′-*cis*-isomer by the typical absorbance at 296 nm. (**b**) Percentage of 9,15,9′-tri-*cis*-ζ-carotene isomer was significantly increased in the cells expressing the CsD27-3 enzyme. A designation of ** = *p* < 0.01 (*t*-test). *n* = 3 independent biological replicate experiments. Error bars represent SD. (**c**) HPLC analyses of carotenes extracted in the absence (pThio-θ) and presence (pThio+CsD27-3) of CsD27-3. MaxPlot chromatograms showing each peak at its spectra are presented. Peak I: 15-*cis*-neurosporene; peak II: 13-*cis*-neurosporene; peak III: 9-*cis*-neurosporene, and peak IV: all-*trans*-neurosporene. Absorption spectra of specific peaks are presented in boxes. (**d**) Percentage of all *cis*-isomers was significantly increased in the cells expressing the CsD27-3 enzyme. Statistical analysis was performed using the Student *t*-test. A designation of *** = *p* < 0.001 (*t*-test). *n* = 3 independent biological replicate experiments. Error bars represent SD.

Furthermore, *CsD27-3* was expressed in zeaxanthin-, β-carotene-, lycopene-, neurosporene- and ζ-carotene-accumulating *E. coli* strains in order to investigate the activity of the encoding enzyme. The introduction of *CsD27-3* in ζ-carotene-accumulating *E. coli* cells growing in dark conditions led to an increase in the *cis*:all-*trans*-ζ-carotene ratio compared to the control strain transformed with the void plasmid, specifically an increase in the content of 9,15,9’-tri-*cis*-ζ-carotene was observed (Figure 7a,b). The construct pThio+CsD27-3 was transformed in *E. coli* cells producing neurosporene growing in dark conditions. An increased ratio of the *cis*:all-*trans*-neurosporene was obtained compared to the control strain transformed with the void vector (Figure 7c,d). The observed isomers have the followingλmax: isomer I: 330,412,436,465; isomer II: 320, 332, 410, 436, 462; isomer III: 330, 410, 435, 462, and IV: 416, 440, 470. These isomers were tentatively identified as 15-*cis*-neurosporene, 13-*cis*-neuropsorene, 9-*cis*-neurosporene, and all-*trans*-neurosporene [34]. The expression of *CsD27-3* in lycopene-accumulating *E.coli* cells unraveled a less pronounced activity, resulting in a relative increase in the content of only one *cis*-isomer, which presumably corresponded to 13-*cis*-lycopene (peak III) (Figure S7a–b). In contrast to ζ-carotene-, neurosporene- and lycopene-accumulating strains, we did not observe any isomerization activity upon the expression of *CsD27-3* in β-carotene and zeaxanthin background (Figure S7c–d). The assays were also done under constant light (Figure S8), but the activity of CsD27-3 could not be detected over ζ-carotene, nor neurosporene.

## 3. Discussion

The physiological role of D27 with respect to its shoot branching and gene regulation has been characterized in several plant species, including rice [9], *Medicago truncatula* [35,36], *Arabidopsis thaliana* [10], chrysanthemum [37,38], and *Fragaria vesca* [30]. In saffron we have identified a D27 orthologous gene, named *CsD27-1*, and two additional *D27-like* genes, *CsD27-2* and *CsD27-3*, with unknown function [39]. All the identified genes encode for proteins with a putative plastid location signal. However, the expression levels of the identified genes in saffron was tissue-specific, with *CsD27-1* and *CsD27-3* showing clearly different expression patterns. *CsD27-1* was highly expressed in the stigma tissue, more specifically in undeveloped stigmas, reaching peak expression in red stigmas, followed by high expression in the root tissue, although SLs have been detected in the roots of saffron but not in the stigma [8]. Interestingly, *CsCCD7* and *CsCCD8* transcripts have been mainly detected in the immature stigma [8], where they can be involved in the elongation of this tissue during its development, controlled by external factors such as temperature and light [29]. The expressions of *CsD27-2* and *CsD27-3* were higher in leaves than in the other tissues. The preferential expression of *CsD27-3* in leaves suggests a specific role in chloroplasts. Further, the *CsD27-3* transcript exhibited large daily oscillations, indicating that *CsD27-3* expression was regulated by the circadian clock. Besides its high expression in leaves, *CsD27-2* transcript levels were also high in the stigma tissue and followed a similar pattern of expression to *CsD27-1*. Expression levels of canonical *D27* in other plant species showed expression in roots and shoots, e.g., rice *D27*, which is especially expressed in the vasculature of these tissues [9]. In Arabidopsis, *D27* expression was developmentally controlled, with high expression levels in the primordia of lateral roots of seedlings, in radicles of immature seeds, and in the style and stigma tissues during the reproductive stage [13]. In root tissue, *CsD27-1* gene expression increased in mycorrhizal roots, as previously observed in rice [40] and in *Lotus japonicus* [41]. Expression analysis showed that *AtD27-like-1* (NP_564838), the homolog of *CsD27-3*, was highly expressed in cotyledons, young intermediate leaves and sepals [11], and the expression levels were 10-fold higher compared to the other *AtD27-like* (NP_680560), the *CsD27-2* homolog, which showed high expression levels in cotyledons, young, intermediate and mature leaves, silique pedicel, and sepals [11]. In the case of rice, the *CsD27-3* homolog in *Oryza rufipogon* was expressed specifically in green tissue and induced by light [42], as in the case of *CsD27-3*. In addition, the analyses of a specific transcriptome of *A. thaliana* plants under high-intensity light without heat stress revealed that *AtD27-like-1* (NP_564838) was present among the 560 upregulated genes regulated by light, and showed an increased expression under high light stress conditions [43]. Further, this protein has been identified in the thylakoid membrane [44]. More recently, it was reported [45] that photosynthesis is gradually established from coleoptile, to incomplete leaf, to complete leaf in rice, but is fully functional in cotyledons of *A. thaliana*. From the published transcriptomes of this work, we analysed the expression of *D27* and *D27-like* genes in rice and Arabidopsis, and observed increased expression levels of the *CsD27-3* homolog in rice (Figure S9) associated with increased expression levels of chloroplast proteins in rice [45], suggesting the involvement of D27-3 in chloroplast development and in the establishment of photosynthetic machinery. By contrast, *D27* and the other *D27-like* (2) gene expression levels were not associated with the establishment of the photosynthesis machinery in rice leaves (Figure S9). The involvement of the protein encoded by the Arabidopsis (At1g64680) homolog to the *CsD27-3* gene in photosynthesis was recently reported in a study looking for NFU3 (Chloroplastic maturation factor) targets [46]. Among other affected proteins were found PsaA, PsaB, PsaC of photosystem I, HCF1, and PETC, all of them characterized by the presence of Fe-S clusters. These clusters are essential for many metabolic processes occurring in chloroplasts, e.g., carbon fixation, sulphur and nitrogen assimilation, amino acid, and vitamin and pigment biosynthetic pathways [47]. NFU3 plays major roles in the biogenesis of chloroplastic 4Fe-4S clusters [48], suggesting the presence of this kind of cluster in D27-3. Interestingly, OsD27 contains iron [9,49], and its activity was inhibited by the presence of silver acetate, which indicated the involvement of an 4Fe-4S cluster in the catalysis [50]. A total of eight conserved cytosines were found in all CsD27 proteins, which can be involved in the coordination of the 4Fe-4S cluster. In addition, a recent publication includes Arabidopsis D27 and D27-like proteins as Fe-containing proteins [51].

For the expression analyses of the *D27* homologs in different tissues of *O. sativa*, available databases of gene expression were explored to investigate the expression of *D27 like* genes (https://rapdb.dna.affrc.go.jp, accessed on 5 February 2022). Data were only obtained for the *CsD27-3* homolog (Os08g0114100, XP_015648697.1), which was expressed in green tissue, induced by light and developmentally regulated. No expression data were found for the *CsD27-2* homolog. Several tomato gene expression databases were also analysed (Figure S10). The tomato *D27* gene was preferentially expressed in roots. The *CsD27-2* homolog (SolyC09g065750.2) showed higher expression levels than *D27* and was mainly expressed in mature leaves, whereas the *CsD27-3* homolog (SolyC06g084610.2) was mainly expressed in young green tissues. In summary, all the obtained and collected data point out the involvement of CsD27-3 and homologs into processes related to chloroplast development.

An unidentified apocarotenoid signal generated during acyclic *cis*-carotene biosynthesis has been shown to regulate nuclear gene expression and chloroplast biogenesis in Arabidopsis tissues [52]. Previous activity assays in carotenoid-accumulating *E. coli* strains performed with OsD27 and AtD27 demonstrated that both enzymes are the carotene isomerases required to initiate SL biosynthesis [7,13,31]. We found evidence that CsD27-1 catalyzes the isomerization of *trans*-β-carotene to *cis*-β-carotene, being the functional homolog of the rice and Arabidopsis D27 isomerase. In addition, transient expression of *CsD27-1* in *N. benthamiana* showed an increase not only in *cis*-β-carotene, but also in those from lutein and zeaxanthin, although in minor proportions. Unexpectedly, we could not detect the isomerase activity in zeaxanthin using the bacterial system, suggesting the absence of appropriate conditions to act in this hydroxylated carotenoid. Interestingly, apocarotenoid products resulting from the expected activity for CCD7 [33], including those derived from β-carotene and zeaxanthin cleavage, increased as a result of the expression of *CsD27-1* in *N. benthamiana*. Furthermore, ABA levels were also increased as observed previously in plants overexpressing *D27* [15]. We also demonstrated, for the first time, the activity of CsD27-3 in ζ-carotene and neurosporene. Upon expression of CsD27-3 in an *E. coli* strain producing ζ-carotene, we observed an increase in 9,15,9’-tri-*cis*-ζ-carotene content, indicating an isomerization activity of CsD27-3 at the 15 double bond. When *CsD27-3* was introduced in a strain producing neurosporene, it led to an increase in three isomers, tentatively identified as 15-*cis*-neurosporene, 13-*cis*-neurosporene and 9-*cis*-neurosporene. The in vivo test unraveled a weak activity promoting an increase in one lycopene isomer that was tentatively identified as 13-*cis*-lycopene [53]. The activity showed by CsD27-3 and its expression patterns could be associated to different processes occurring during the early development of the chloroplast. On one hand, *cis*-carotenes have been reported to be resistant to non-enzymatic degradation [54]. Therefore preservation of *cis*-configuration could be a way to preserve the small pool of carotenoids in the initial steps of chloroplast development, and a guarantee of good photosynthetic development [55,56]. On the other hand, *cis*-carotene cleavage products have been linked to feedback and feedforward regulation of transcriptional and translational processes, as well as to organelle and nucleus communications [57]. Another point to take into consideration is that recently, an apocarotenoid signal, probably originating from *cis*-neurosporene and perhaps from di-*cis*-ζ-carotene, has been shown to be involved in the regulation of chloroplast biogenesis and leaf morphology in Arabidopsis [58], although the presence and function of this apocarotenoid requires further investigation in other plant species.

In conclusion, this study illustrates the differential activities of CsD27-1 and CsD27-3 enzymes and reveals for the first time the isomerase activity of CsD27-3. Whether CsD27-3 activity provides a substrate for the production of *cis*-apocarotenoids during the day/night cycle, influencing metabolic and morphological traits according to light quality, or participates in the maintenance of *cis*/*trans* homeostasis in chloroplast, will require additional and more in-depth research.

## 4. Materials and Methods

### 4.1. Plant Material and Treatments

*C. sativus* cultivated in the Jardín Botánico de Castilla-la Mancha (Albacete, Spain) were used for all the experiments. Corms, leaves, roots, stamens, tepals and stigmas were collected at different developmental stages as previously described [59]. All the tissues were frozen in liquid nitrogen and stored at −80 °C until further use.

Mycorrhizal infection and detection were performed as previously described [28].

### 4.2. Isolation of cDNA Sequences Encoding D27 Enzyme in Saffron

*C. sativus* roots, leaves and orange stigmas were used for total RNA extraction using a RNeasy Plant Mini Kit (Qiagen, Hilden, Germany). The obtained RNA was treated with DNase and quantified. For all the samples, 1 µg was used for first-strand cDNA synthesis by reverse transcription (RT) using a First-strand cDNA Synthesis Kit (GE Healthcare Life Sciences, Buckinghamshire, UK). The cDNAs were used as templates for amplification of D27 genes from saffron using PCR primers designed from the sequences obtained from previous saffron transcriptomes [18,29] (Table S1). The conditions for PCR were as follows: 2 min at 95 °C, 35x (20 s at 95 °C, 20 s at 60 °C and 2 min at 72 °C) and finally 5 min at 72 °C. The PCR products were separated in 1.0% agarose gels stained with ethidium bromide, purified, ligated into the pGEMT vector (Promega, Madison, WI, USA) and introduced into *E. coli* cells.

### 4.3. Phylogenetic Analysis

The amino acid sequences were aligned using the BLOSUM62 matrix with the ClustalW (http://www.clustal.org, accessed on 2 February 2022) algorithm-based AlignX module from MEGA Version 7.0 [60] (http://www.megasoftware.net/mega.html, accessed on 2 February 2022), and used to generate a Neighbor-Joining tree with bootstrap support (2500 replicates). Gaps were deleted pairwise.

### 4.4. DNA Sequencing and Analysis of the DNA and Protein Sequences

Sequencing reactions were carried out using an automated DNA sequencer (ABI PRISM 3730xl, Perkin Elmer, Macrogen Inc., www.macrogen.com, accessed on 2 May 2021). Similarity searches were done using the BLAST suite of programs of the National Center for Biotechnology Information (NCBI; http://www.ncbi.nlm.nih.gov, accessed on 2 May 2021). Motif searches were performed using SignalP (http://www.cbs.dtu.dk/services/SignalP, accessed on 2 August 2021). The proteins were modeled using the Phyre server (http://www.sbg.bio.ic.ac.uk/phyre2, accessed on 21 November 2021).

### 4.5. Expression Analysis

For expression analyses, stigmas, corms, roots, leaves, stamens and tepals were dissected from ten plants and the same kind of tissues were pooled. Three independent pools of these tissues were used as three biological replicas. TRIzol reagent (Invitrogen) was utilized for RNA extraction. 1 µg of total RNA was used for first-strand cDNAs by reverse transcription (RT) using an oligo dT primer and a First-strand cDNA Synthesis Kit (GE Healthcare Life Sciences, Buckinghamshire, UK) according to the manufacturer’s instructions. The cDNAs were used as templates using gene-specific primers (Table S1). The cycling parameters of qPCR consisted of an initial denaturation at 94 °C for 5 min, 40 cycles at 94 °C for 20 s, 58 °C for 20 s, 72 °C for 20 s, and a final extension at 72 °C for 5 min. The assays were conducted in a StepOne™ Thermal Cycler (Applied Biosystems, Foster City, CA, USA) and analyzed using StepOne software v2.0 (Applied Biosystems, Foster City, CA, USA). DNA melt curves were created for each primer combination in order to confirm the presence of a single product. Gene expression was calculated using the 2^−ΔΔCT^ method. The expression data were normalized based on *CsRP18S* [8,59].

### 4.6. Carotenoid Extraction and HPLC Analysis

Carotenoids were extracted and analyzed from 1 g of lyophilized tissue with methanol:chloroform (1:1, *v/v*), as described previously [61]. The extract was reduced to dryness and kept under a nitrogen atmosphere at −80 °C until HPLC-DAD analysis.

HPLC analysis of carotenoids was carried out using an Agilent 1100 liquid chromatography system equipped with a photodiode array detector. Data were analyzed with Empower Software (Waters, Milford, MA, USA). The dry extracts were redissolved in tert-methyl-butyl-ether and filtered through a 0.45 μm membrane. An aliquot (20 μL) of samples was injected into a HPLC C30 column (column temperature 25 °C, flow rate 1.0 mL/min). Compounds were detected at 450 nm. Carotenoids were identified based on their retention time and absorption spectra compared with authentic standards, and were quantified according to their standard curves as previously described [61].

### 4.7. D27 Activity in E. coli Cells

To generate pThio-Dan1-CsD27-1 and pThio-Dan1-CsD27-3, the *CsD27* cDNAs were amplified without the plastid transit peptide (Table S1) and cloned by recombination using an In-Fusion^®^ HD Cloning Plus CE kit (Clontech Laboratories, Inc., Mountain View, CA, USA) into the plasmid pThio-Dan1 flanked by EcoRI restriction enzyme. The resulting plasmids were used for expression in carotenoid accumulating *E. coli* cells. Positive colonies from the transformation reaction were inoculated in 5 mL of 2x YT media containing the antibiotics ampicillin (50 μg/mL) and chloramphenicol (25 μg/mL) and grown overnight at 30 °C at 190 rpm. The overnight cultures were used to inoculate 50 mL 2x YT and cultured at 30 °C until an optical density of 0.8 at 600 nm (OD600) was reached. The cells were induced with 0.8% arabinose and grown overnight at 20 °C. The cells were harvested by centrifugation (6000 rpm for 10 min), and the pigments were repeatedly extracted with a total volume of 10 mL of acetone until the pellet was colorless. The solvent was evaporated under N_2_ gas, and the pigments were resuspended with 0.3 mL tert-methyl-butyl-ether. After centrifugation (13,000 rpm for 10 min), the extracts were analyzed using HPLC as previously described [62].

### 4.8. Transient Expression of CsD27-1 in Nicotiana benthamiana

The pDGB3Ω1: [(PNos: Hyg: TNos): (P35S:CsD27-1:T35S)] was constructed following the GB4.0 assembly strategy [63]. Briefly, the first step was the domestication of *CsD27-1* by removing the internal BsaI and BsmBI sites and adding the adapters; PCR amplifications of *CsD27-1* using GB-adapted primers, designed by GB4.0 tools (https://gbcloning.upv.es, accessed on 15 March 2019), were performed using pGEMT-CsD27-1 as a template. The resulting PCR fragment was cloned into the pUPD2 vector to yield domesticated GB parts using a BsmBI restriction-ligation reaction. Several assembly construct combinations were performed via restriction-ligation reactions in order to obtain the final construct which was transformed into *E. coli*. Positive white clones were selected under spectinomycin (50 µg/mL) for the pDGB3Ω1 construct, and further confirmed by digestion and sequencing using an automated DNA sequencer (ABI PRISM 3730xl, Perkin Elmer, Macrogen Inc., Seoul, Korea). *Agrobacterium tumefaciens* strain GV3101 was transformed by electroporation with the construct and selected on YEB agar with the corresponding antibiotics. Transient expression experiments were carried out in *N. benthamiana* leaves as previously described [64]. As controls, transformations of leaves with the empty vector were performed. Eight-days after agroinfiltration, the leaves were collected and lyophilized. The metabolites extracted with acetone and carotenoids were analyzed by HPLC-DAD, as previously described [61]. Three independent experiments were performed to generate three biological replicates for carotenoid analysis.

## Data Availability

The original contributions presented in the study are included in the article/Supplemental Material. Further inquiries can be directed to the corresponding authors.

## Supplementary Materials

The following supporting information can be downloaded at: https://www.mdpi.com/article/10.3390/ijms231810543/s1.
