# Supplementary material for: Subfunctionalization of D27 Isomerase Genes in Saffron
Source: Int J Mol Sci. 2022 Sep 11;23(18):10543. doi: 10.3390/ijms231810543 (PMC9504799; doi:10.3390/ijms231810543)
Supplement: Supplementary file 1 [file ijms-23-10543-s001.zip › ijms-1881812-supplementary.pdf]

Supplemental Figures and Tables

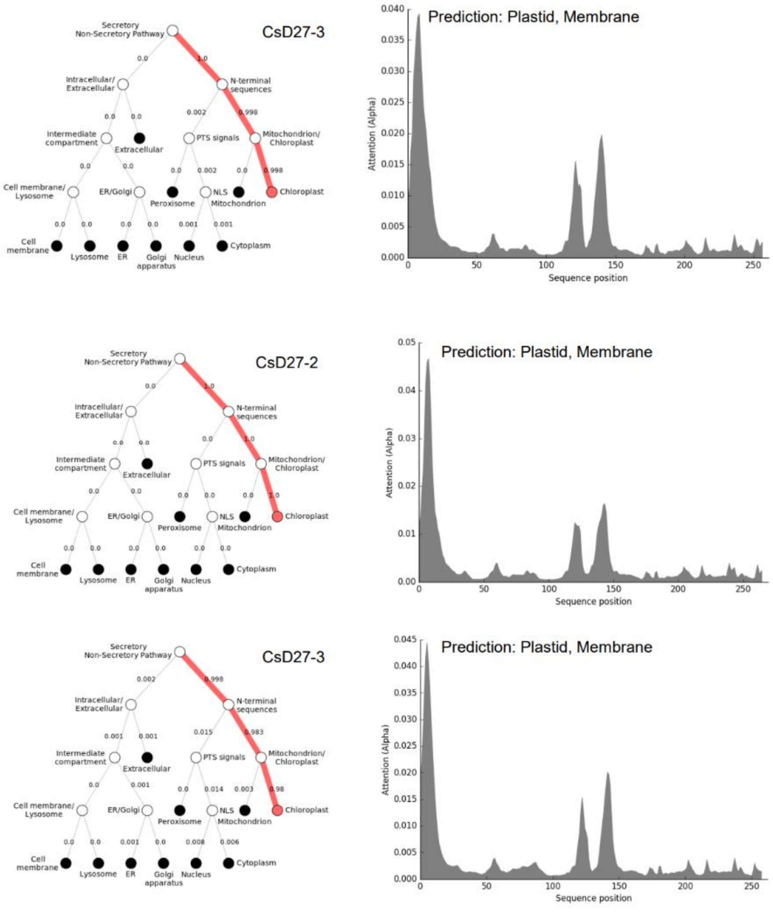

Supplemental Figure S1. Prediction of subcellular location of CsD27 proteins, by using: https://services.healthtech.dtu.dk/service.php?DeepLoc-1.

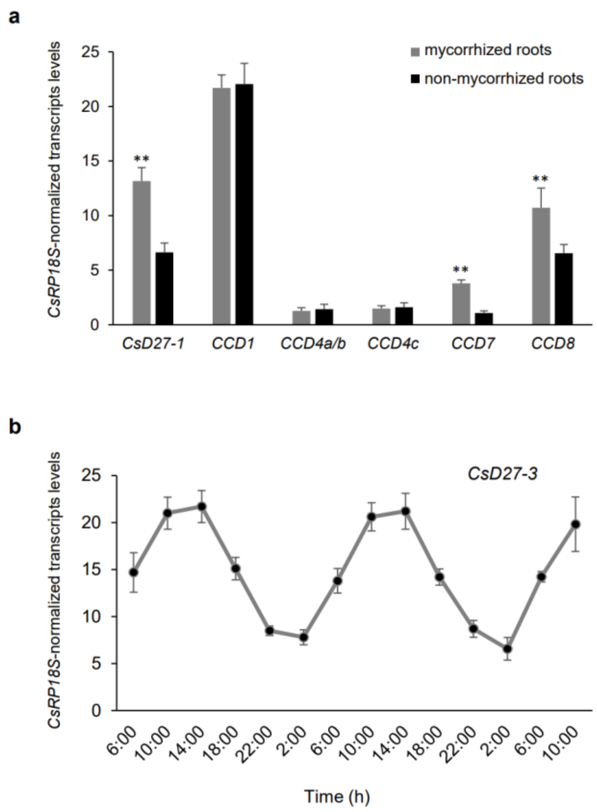

Supplemental Figure S2. Characterization of expression of *CsD27-1* and *CsD27-3* in root and leaf tissue, respectively. (**a**) Expression levels of *CsD27-1* and other *CCD* genes, previously characterized in saffron, both in mycorrhized and non-mycorrhized roots. Statistical analysis was performed using. Asterisks indicate statistical significance (** =p < 0.01 according a pairwise t-test). n = 3 independent biological replicate experiments. Error bars represent SD. (**b**) Expression levels of *CsD27-3* in leaves collected at different time points. Data were presented as means ± SD of three biological replicates.

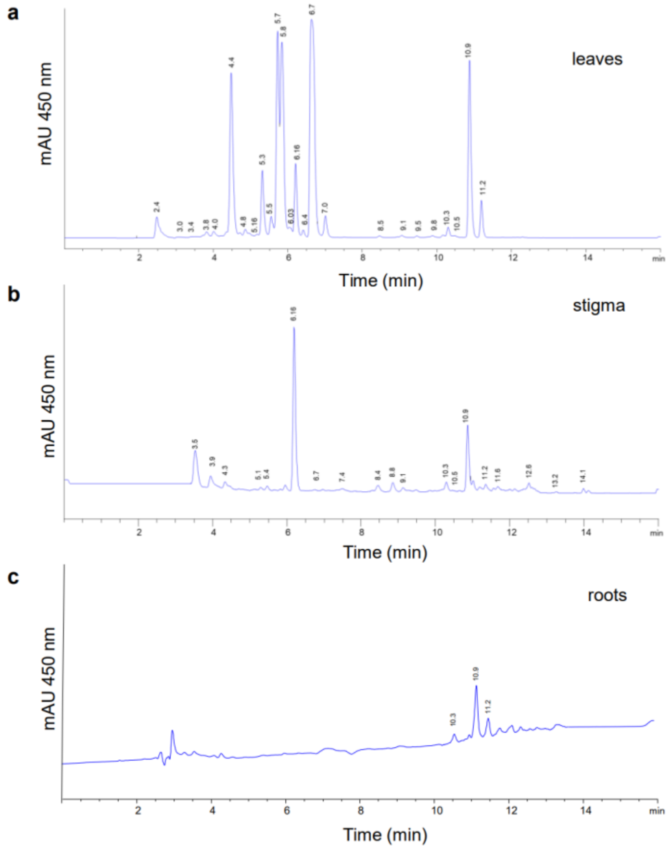

Supplemental Figure S3. Representative chromatograms at 450 nm for carotenoid analyses in saffron tissues. (**a**) Representative chromatogram of green leaves. (**b**) Representative chromatogram of red stigmas. (**c**) Representative chromatogram of roots. Numbers correspondence is present in Supplemental Table 2.

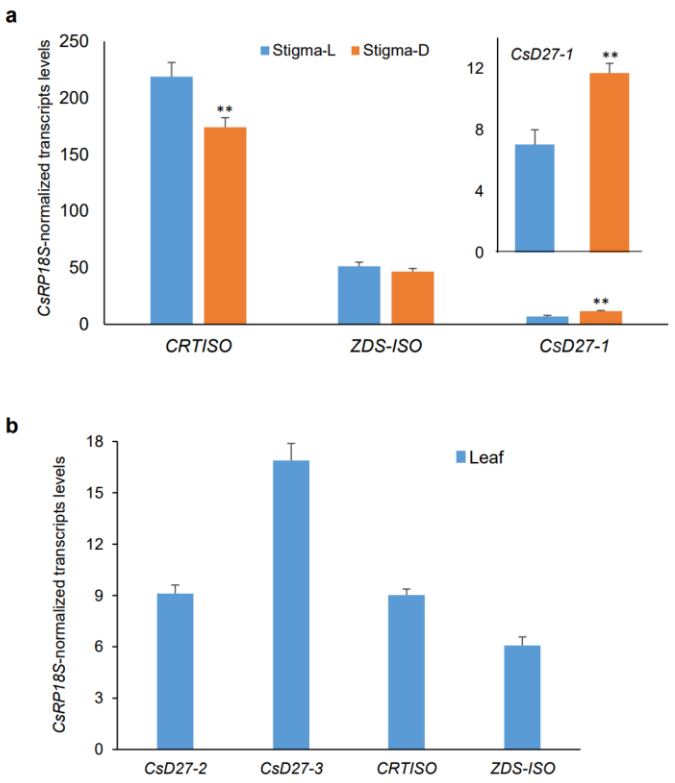

Supplemental Figure S4. Transcript levels of carotenoid isomerase genes in stigma (at dark (D) and light (L) conditions) (**a**) and leaf tissue (**b**), as determined by qRT-PCR. Statistical analysis was performed Using. Asterisks indicate statistical significance (** =p < 0.01 according a pairwise t-test). N = 3 independent biological replicate experiments. Error bars represent SD.

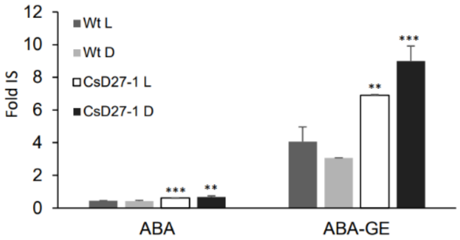

Supplemental Figure S5. levels of ABA and ABA glucosyl ester (ABA-GE) in *N. benthamiana* leaves agroinfiltrated with 35S::CsD27-1 or 35S::θ in both dark (D) and under standard light conditions (L). Statistical analysis was performed using Asterisks indicate differences from wild type (** = $p < 0.01$, *** = $p < 0.005$ (t-test)). N=3 independent biological replicate experiments. Error bars represent SD.

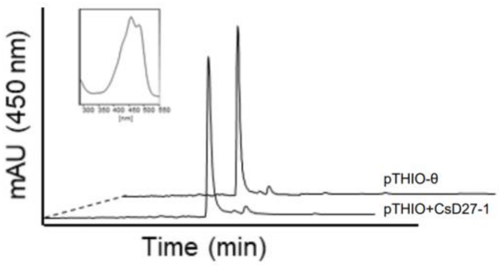

Supplemental Figure S6. HPLC analysis of CsD27-1 activity on *E. coli* cells producing zeaxanthin. No isomerase on zeaxanthin activity was found in cells expressing pThio+CsD27-1.

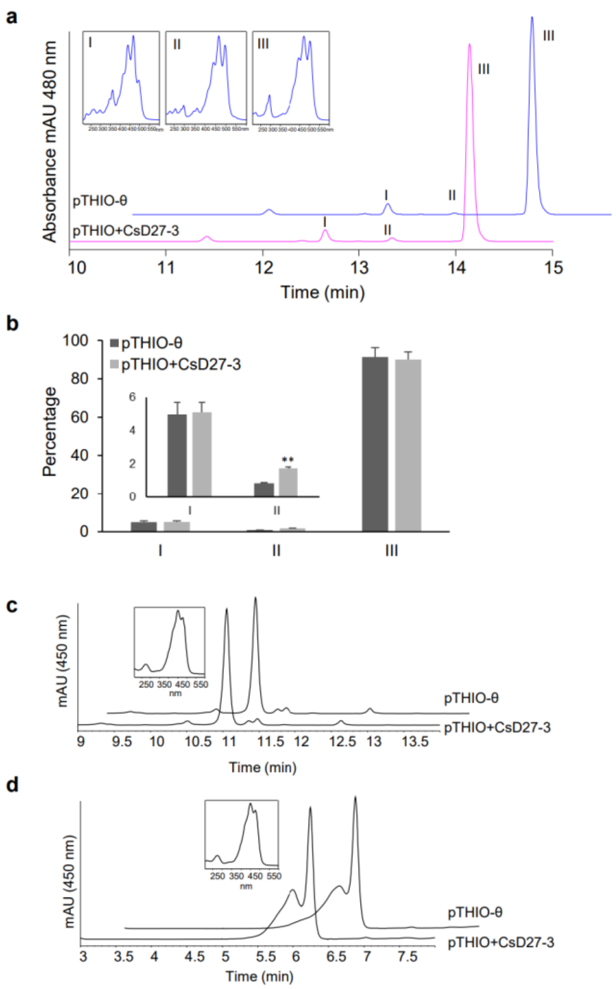

Supplemental Figure S7. HPLC analysis of *in vivo* CsD27-3 activity under dark conditions, and carotenoids accumulating *E. coli* cells transformed with pThio +CsD27-3 and the corresponding empty plasmid (pThio-θ). (**a**) CsD27-3 enzyme effects on all-trans-lycopene (III) and two different isomers:15-cis-lycopene (I) 13-cis-lycopene (II). (**b**) Percentage of only one cis isomer (II) was significantly increased in the presence of CsD27-3 enzyme. A designation of ** =p < 0.01 (t-test), n = 3 independent biological replicate experiments. Error bars represent SD. (**c**) No isomerase activity on β-carotene was found in cells expressing pThio+CsD27-3. (**d**) No isomerase activity on zeaxanthin was found in cells expressing pThio+CsD27-3.

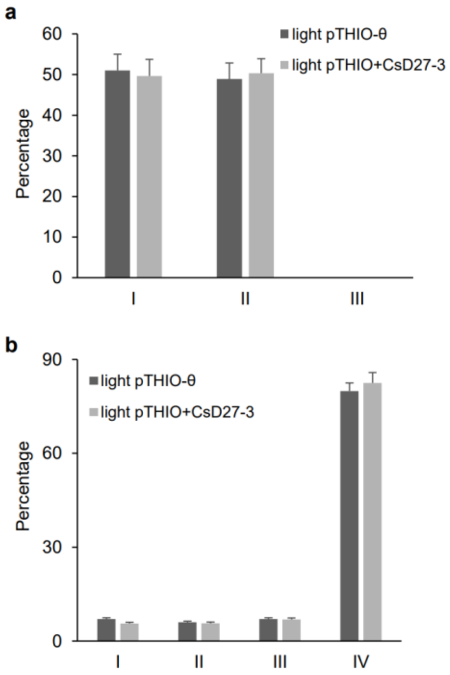

Supplemental Figure S8. Analysis of *in vivo* CsD27-3 activity under light conditions. Percentage of ζ-carotene and neurosporene accumulating *E. coli* cells transformed with pThio+CsD27-3 and the corresponding empty plasmid (pThio-θ). (**a**) CsD27-3 enzyme effects on 9-*cis*-ζ-carotene (III); 9,15,9'-tri-*cis*-□ -carotene (I) and 9,9'-di-*cis*-ζ-carotene (II). (**b**) CsD27-3 enzyme effects on neurosporene. N = 3 independent biological replicate experiments. Error bars represent SD. Non-significant differences were found (P < 0.05)(t-test).

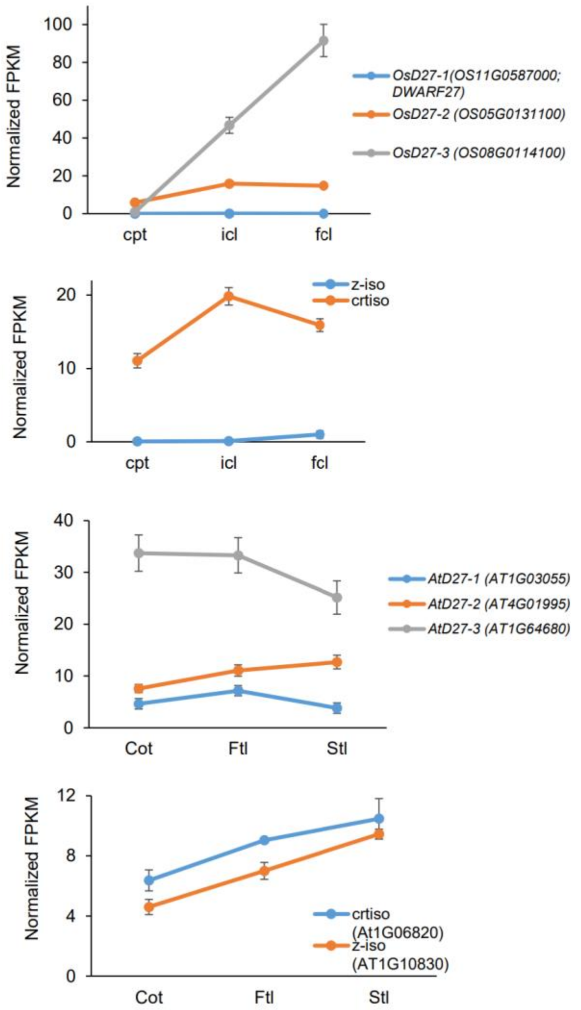

Supplemental Figure S9. RNA-seq-based gene expression values (FPKM) of *D27* and *D27-like* genes in rice and Arabidopsis. Data was presented as the means ± SD of three biological replicates. Coleoptile (cpt) before the incomplete leaf (icl) appeared in rice andbcotyledon (cot) before the first true leaf (ftl) appeared in Arabidopsis as stage 1
(S1), (icl) before the first complete leaf (fcl) appeared in rice and ftl before the second true leaf (stl) appeared in Arabidopsis as stage 2, fcl in rice and stl in Arabidopsis appeared fully as stage 3. Data obtained from Shi et al., (2020).

SolyC09g065750.2 (CsD27-1 homolog)

Experiment: D006: Transcriptome analysis of various tissues in wild species *S. pimpinellifolium*, LA1589

ID	genotype	sample	normlized expression (RPKM)
S0608	LA1589	anthesis flowers (0DPA)	0.12
S0609	LA1589	10 days post anthesis fruit (10 DPA1)	0.08
S0610	LA1589	10 days post anthesis fruit 2 (10 DPA2)	0.08
S0611	LA1589	20 days post anthesis fruit (20DPA)	0.04
S0612	LA1589	rippening fruit (33DPA)	0
S0613	LA1589	cotyledons (COTYL)	0.71
S0614	LA1589	hypocotyl (HYPO)	1.11
S0615	LA1589	vegitative meristems (MERI)	0.05
S0616	LA1589	mature leaves (ML)	0.14
S0617	LA1589	whole root (ROOT)	12.69
S0618	LA1589	young flower buds (YFB)	0
S0619	LA1589	young leaves (YL)	0

SolyC08g008630.2 (CsD27-2 homolog)

Experiment: D006: Transcriptome analysis of various tissues in wild species *S. pimpinellifolium*, LA1589

ID	genotype	sample	normlized expression (RPKM)
S0608	LA1589	anthesis flowers (0DPA)	78.73
S0609	LA1589	10 days post anthesis fruit (10 DPA1)	31.12
S0610	LA1589	10 days post anthesis fruit 2 (10 DPA2)	26.24
S0611	LA1589	20 days post anthesis fruit (20DPA)	14.48
S0612	LA1589	rippening fruit (33DPA)	4.97
S0613	LA1589	cotyledons (COTYL)	85.1
S0614	LA1589	hypocotyl (HYPO)	35.37
S0615	LA1589	vegitative meristems (MERI)	23.88
S0616	LA1589	mature leaves (ML)	147.25
S0617	LA1589	whole root (ROOT)	1.43
S0618	LA1589	young flower buds (YFB)	23.35
S0619	LA1589	young leaves (YL)	25.2

SolyC06g084610.2 (CsD27-3 homolog)

Experiment: D006: Transcriptome analysis of various tissues in wild species *S. pimpinellifolium*, LA1589

ID	genotype	sample	normlized expression (RPKM)
S0608	LA1589	anthesis flowers (0DPA)	11.13
S0609	LA1589	10 days post anthesis fruit (10 DPA1)	8.41
S0610	LA1589	10 days post anthesis fruit 2 (10 DPA2)	6.37
S0611	LA1589	20 days post anthesis fruit (20DPA)	7.09
S0612	LA1589	rippening fruit (33DPA)	3.32
S0613	LA1589	cotyledons (COTYL)	32.43
S0614	LA1589	hypocotyl (HYPO)	21.01
S0615	LA1589	vegitative meristems (MERI)	50.13
S0616	LA1589	mature leaves (ML)	39.21
S0617	LA1589	whole root (ROOT)	7.89
S0618	LA1589	young flower buds (YFB)	56.93
S0619	LA1589	young leaves (YL)	53.49

Supplemental Figure S10. Normalized expression data of *D27* and *D27-like* genes from tomato in different tissues. Red frames indicate the higher expression values among the tissues tested.

Supplemental Table S1. Oligonucleotide sequences used for cloning, expression analyses and functional characterization.

	Forward 5'-3'	Reverse 5'-3'
Cs-D27-1-cloning	ACCTTTATGGAATCCAGTCTTCT	TGCTGCTTCTAGTTTGAACTGGA
Cs-D27-2-cloning	AGTTATCCTCATGGAAGCGGCTCT	TGTTACACCTTTGGACACTGAG
Cs-D27-3-cloning	ATGGAGCTTTTACCAATGCCCA	CTATACATCTATAATCTCTCTCTG
Cs-D27-1-expression	GCACCAATTGTGTTGGTCTG	CGTCTTCAGGTTCAGGTGGT
Cs-D27-2-expression	AAGTTCCGGGTACTCTGCTC	GCTTCCTCAGTCTCCGAGTT
Cs-D27-3-expression	ATGGAGCTTTTACCAATGCCCA	CAACACATCCGCTGTTCTCCA
Cs-D27-1-pTHIO	cgcccttggcgaattcATGACGGAGTACAATGATAACTG	taccctcgaggaattcCTAGTTTGAACTGGAGCAAT
Cs-D27-2-pTHIO	cgcccttggcgaattcATGAATCACTATAGAAAAAATTG	taccctcgaggaattcTTACACCTTTGGACACTGAG
Cs-D27-3-pTHIO	cgcccttggcgaattcATGTCACCTCCATACCAAGTTCG	taccctcgaggaattcCTATACATCTATAATCTCTCTCTG
CsCRTISO	GGAGGAGGAATTCGACGCTA	TGACCAACTGCTTCCAATGC
CsZETA-ISO	AAGTCTCCGAGATGTAGGCG	AGGTGGAGGGGTAAAGAAGC

Supplemental Table S2. Chromatographic and spectroscopic characteristics of the carotenoids isolated from stigma, leaves and root tissues.

Retention time	λmax	compound	tissue
2.4	(320), 433 ,458	unidentified	L
2.9	430, 454	trans-crocetin	S
3.05	375, 397, 420	unidentified	L
3.5	400sh, 424, 450	unidentified	S
3.4	418, 440, 467	trans-neoxanthin	L
3.8	(315,330) 406, 430, 456	di-Z-violaxanthin	L
3.9	434, 458	unidentified	S
4.0	416, 440, 470	trans-violaxanthin	L
4.3	442, 467	unidentified	S
4.4	(328), 414, 438, 466	cis-9-violaxanthin	L
4.8	400, 422, 448	luteoxanthin isomer	L
5.1	430	unidentified	S
5.16	(350), 420sh, 440, 468	13-Z-lutein	L
5.3	420, 444, 472	Lutein isomer	L
5.4	458	unidentified	S
5.5	(331), 418sh, 438, 466	13′-Z-lutein	L
5.7	Chlorophyll b		L
5.8	422, 446, 472	all-E-lutein	L
5.9	(328), 421, 444, 468 cis	13-Z-zeaxanthin	L
6.03	chlorophyll		L
6.16	450, 476	all-E-zeaxanthin	L, S
6.7	Chlorophyll a		L
6.73	394, 416, 445	luteoxanthin	S
7.0	chlorophyll		L
7.4	445	15-Z-cryptoxanthin	S
8.4	426sh, 450, 475	β-cryptoxanthin	S
8.5	444, 478	unidentified	L
8.8	406 sh 428, 454 and phytoene	unidentified	S
9.1	420sh, 446, 472	unidentified	L, S
9.5	428, 452	unidentified	L
9.8	chlorophyll		L
10.3	444, 468	α-carotene	L, S
10.5	(338), 442, 466	cis-13-β-carotene	L, S
10.9	451, 477	all-trans-β-carotene	L, S, R
11.2	(342), 446, 472	cis-9-β-carotene	L, S, R
11.6	428, 454, 488	δ-carotene	S
12.6	440, 464, 492	γ-carotene	S
13.2	(369), 440, 466, 496	Cis-9-lycopene	S
13.9	472, 502	all-trans-lycopene	S
14.1	(363), 446, 472, 503	cis-lycopene	S

S= stigma; L= leaf, R= Root
